# Public health and cancer in Latin America: the region does not lack evidence; it lacks effective state capacity, enforceable laws, sustained prevention, and early diagnosis

**DOI:** 10.3389/fpubh.2026.1812602

**Published:** 2026-05-13

**Authors:** Aleixandre Brian Duche-Pérez, Alberto Vittorio Camargo-Riega, Renato Paredes-Velazco

**Affiliations:** 1 Department of Anthropology, National University of San Agustín, Arequipa, Peru; 2 Faculty of Law and Political Science, Catholic University of Santa María, Arequipa, Peru

**Keywords:** cancer, health inequities, health system fragmentation, state capacity, sustained prevention

## 1 Introduction

In Latin America and the Caribbean, cancer can no longer be framed only as a clinical problem. It constitutes a public health emergency because, from the first point of contact with the health system, it exposes who can reach timely care and who falls behind. The regional burden is substantial. In 2022, the region recorded 1,551,060 new cases, 749,242 deaths, and a five-year prevalence of 4,096,032 people living with cancer (1). Yet the most consequential question extends beyond how many people develop cancer to how much health systems lose to preventable delays. Cancer becomes more lethal when institutions convert clinical time into administrative time, and that lost time does not fall equally across populations (1).

This regional framing must also remain analytically precise. Latin America and the Caribbean form a heterogeneous subregion of the Americas, marked by linguistic, cultural, territorial, and institutional diversity, but connected by persistent challenges in cancer control and public health governance. Specialized literature already recognizes Latin America and the Caribbean as a relevant field of analysis in cancer control (2). At the same time, recent comparative evidence available for this discussion is reported mainly at the level of South and Central America rather than through country-disaggregated analyses across all Latin American and Caribbean settings (3). This distinction matters because regional arguments should not erase internal variation, even when a shared pattern of inequity remains visible across the subregion.

This inequality does not emerge at the end of the care pathway. It emerges at the beginning. Across the region, recurring patterns mark health inequity: insufficient or intermittent screening coverage, limited awareness of warning signs among the public and health personnel, shortages of specialists and diagnostic infrastructure, high out-of-pocket costs, and cumulative delays across appointments, tests, biopsies, and pathology reports (1). From a public health perspective, these patterns do not reflect only “late” individual choices. They reflect social and territorial gradients that shape the real capacity to prevent, detect, and treat disease. In other words, when people arrive late, they rarely arrive late by chance. They arrive late because layered social, geographic, and administrative barriers overlap and compound (4, 5).

Recent global evidence reinforces why this argument should be read as a public health rather than a purely clinical concern. Latin America appears explicitly in the geographic distribution of treatable cancer mortality, and prostate cancer accounts for the highest number of treatable deaths in most countries of the region. In South America, 214,800 deaths within 5 years of diagnosis were estimated to be avoidable, representing 43.8% of expected cancer deaths, with an age-standardized rate of 67.9 per 100,000 inhabitants. Of these, 128,200 were classified as preventable and 86,600 as treatable, while the total expected cancer deaths reached 490,100. In Central America, 56,200 deaths were estimated to be avoidable, representing 47.1% of expected cancer deaths, with an age-standardized rate of 54.6 per 100,000 inhabitants; 29,300 were preventable and 27,000 treatable, out of 119,300 expected deaths overall (3). Although these data do not provide country-by-country estimates for all Latin American settings and do not resolve the Caribbean separately in the material used here, they show that avoidable cancer mortality is already measurable at the subregional level and that it reflects both failures of prevention and failures of timely treatment.

When one examines the region country by country, contexts vary, but the storyline often remains consistent. Health systems fragment services, concentrate capacity in major cities, and route patients through access pathways that penalize those who live far from referral centers or depend on weak networks. In Colombia, research on access shows how administrative barriers, including paperwork, authorizations, referrals, and waiting times, can block or delay the effective use of services even when coverage exists on paper (6, 7). In cervical cancer, a highly preventable disease, this institutional friction carries direct consequences. Health system actors have described coordination tensions and operational failures that weaken control from screening through treatment (8), and narrative reviews on timely detection emphasize persistent barriers that ultimately drive late diagnoses (9). In rural areas, user perceptions within the subsidized regime highlight how distance, transport availability, service supply, and insurance administration function as everyday obstacles (10).

Chile provides a useful contrast because its explicit health guarantees policy, AUGE/GES, aims to structure clinical pathways through defined management standards (11). Even so, public health effectiveness does not follow automatically from guidelines or formal frameworks. It depends on whether systems translate norms into real coverage, timely diagnosis, and continuity of treatment. This tension remains politically uncomfortable. The region has accumulated robust evidence on what works, but the state, understood as stewardship, regulation, efficient financing, and sustained implementation, often remains the weak link (1). This concern is not new in cervical cancer. Early analyses already highlighted structural challenges to a consistent regional response (12), and recent assessments confirm that gaps persist even as technical roadmaps become clearer (13).

The prevention lens sharpens the argument further. Coverage does not depend only on offering screening. It depends on ensuring attendance, follow-up, and timely resolution. In El Salvador, factors shaping screening attendance indicate that demand reflects social conditions, logistical constraints, and prior experiences with the health system, not merely test availability (14). When information systems do not support follow-up and traceability, prevention fails. For this reason, scholars have proposed strengthening screening information systems in developing countries to prevent care from fragmenting into disconnected visits and unresolved results (15). Along similar lines, comparative arguments about cervical cancer control in the region emphasize that regulatory strength and spending efficiency can matter as much as, or more than, nominal investment levels when the goal is to convert resources into effective vaccination coverage, screening, and timely treatment (16). Recent evidence also shows that Mexico's 2014 tax on sugar-sweetened beverages increased the proportion of non-consumers of soft drinks by 14%, illustrating how population-level fiscal policy can modify exposure patterns relevant to cancer prevention (3).

Tobacco control offers a parallel reminder that public health can reduce cancer risk before symptoms or tumors appear. Effective prevention relies less on individual timely diagnosis and more on sustained policy decisions that regulate markets, reduce exposure, and protect populations (17). This point is consistent with recent comparative findings showing that lung cancer accounts for the greatest number of preventable cancer deaths in North and South America, while prostate cancer accounts for the greatest number of treatable cancer deaths in most of Latin America (3). Oral cancer, in turn, reinforces the regional dimension of inequity. Data gaps and underreporting, limited infrastructure, and a substantial burden linked to tobacco use make it essential to align research and public policy through regional collaboration and locally grounded evidence (18).

This broader view strengthens the core message. Avoidable conditions become deadly because systems fail. A common sequence repeats, under different names, across many countries in the region. Someone notices an unusual symptom and delays seeking care because time, money, or transport constraints limit access. Once they enter the system, they face a prolonged journey through referrals, authorizations, waiting lists, and rescheduled tests. In that interval, which often reflects administrative processes more than clinical needs, disease progresses (1). The central thesis follows: in Latin America, cancer worsens less because of missing knowledge and more because of public health failures, including weak governance, intermittent implementation, centralized capacity, and access pathways that punish those who begin at a disadvantage (1, 19).

This article argues that Latin America must treat cancer as a public health priority, not merely a matter of clinical care. It shows how weak state capacity, expressed through fragile legislation, uneven financing, discontinuous prevention, and unreliable pathways to early diagnosis, deepens inequities across the region. It also calls for concrete, sustained measures that strengthen prevention and ensure timely diagnosis as core obligations of public policy.

## 2 Public health framework and relevance (why it matters)

In Latin America and the Caribbean, cancer does not distribute randomly. It concentrates where social, territorial, and political disadvantages accumulate. This premise frames the regional cancer burden as a product of the social determinants of health rather than a simple aggregation of individual diagnoses. Under this framework, risk exposure, early detection, and survival depend on the conditions in which people are born, grow, work, and age, and on how those conditions shape education, employment, social protection, and effective access to services (4, 5, 20–22). This perspective also requires recognizing that reducing health inequalities demands action not only on living conditions but also on the unequal distribution of power, money, and resources that structures those conditions (23). Accordingly, prognosis depends not only on tumor biology but also on whether people can complete timely pathways of prevention, diagnosis, treatment, follow-up, survivorship, and palliation (1, 24).

Latin American inequity extends beyond income. It operates as structural inequality because it combines precarious employment, informality, environmental exposure, and limited institutional capacity to regulate, enforce, and protect. From a public health perspective, this concern must now be expanded beyond the social determinants of health to include the commercial determinants of health, which are increasingly recognized as powerful drivers of cancer risk (23). These determinants refer to private-sector activities that shape social, physical, and cultural environments through business practices and political influence, thereby structuring exposure to tobacco, alcohol, air pollution, obesity, and physical inactivity, all of which are linked to cancer (23). In this sense, cancer cannot be understood only as an individual biomedical event; it is also the outcome of harmful social, economic, and regulatory environments. This structural reading matters because commercial interests can obstruct public health regulation, as recent international debates on tobacco policy, industry lobbying, and environmental governance have shown (23).

In occupational cancer, this configuration produces disease systematically. Risk increases when informal work and precarious labor weaken prevention and shift the burden toward groups with less power to secure protection. Algranti et al. argue that, across the region, occupational exposures such as ultraviolet radiation in agriculture and construction coexist with adverse social conditions, including precarious housing, inadequate nutrition, pollution, and limited water and sanitation. These conditions intensify risk and undermine primary prevention. A decisive barrier compounds the problem: large shares of informal workers remain outside preventive programs, and many workplaces lack updated exposure information. As a result, health systems could prevent part of the cancer burden, yet societies normalize that burden because labor and regulatory environments impose risk rather than reflecting individual choice (25–28). This is precisely why cancer control requires a structural public health approach and a regulatory environment oriented to collective wellbeing rather than short-term commercial gain (23).

Territorial inequity also produces a concrete, measurable health injustice. Even when a country offers oncology services, the critical question asks whether people reach those services in clinically meaningful time. Across the region, specialized care concentrates in large cities, while rural and peripheral areas require long-distance travel even for basic care. This pattern creates bottlenecks, waiting lists, and delays that worsen prognosis (1). Countries with historically centralized high-complexity resources, such as national cancer institutes or referral hospitals, often reproduce this dynamic, and low connectivity, dispersed populations, and high transport costs amplify it. Under these conditions, coverage without effective accessibility produces a form of “paper access”: the right exists formally, but it does not materialize within clinically useful time.

Centralization does not operate only through geography. It also operates through technology and the health workforce. Shortages of pathologists, oncologists, and other specialists can slow or fragment diagnostic pathways even when patients enter the system (29, 53). For this reason, public health responses cannot rely only on building more hospitals. They must build decentralized, functional networks. In Latin America, proposed solutions to close diagnostic and therapeutic timeliness gaps include telepathology, ambulatory chemotherapy in remote territories, and remote clinical collaboration to sustain continuity of care (1). However, innovation will not reduce inequity if governments concentrate it in capital cities and fail to integrate it into territorial networks with clear referral pathways, stable financing, and trained personnel.

In several Latin American countries, the problem does not stem only from service scarcity. System fragmentation, involving multiple insurers, providers, and administrative steps, turns care trajectories into endurance tests. In Colombia, research describes administrative barriers and access constraints that disproportionately affect vulnerable and rural populations (6, 7, 9, 10, 30). This dynamic becomes especially harmful in oncology because cancer care requires continuity from referral and diagnostic confirmation to treatment initiation and uninterrupted follow-up. When systems fragment care, evidence links fragmentation to worse survival outcomes in cancers such as breast and colorectal (31, 32). The relevance of the cancer continuum is therefore practical as much as conceptual: effective cancer control depends on linking prevention, early detection, diagnosis, treatment, survivorship, and palliative care within one continuous pathway rather than treating them as isolated services (24).

Cervical cancer functions as a marker of inequality in Latin America. Although it is highly preventable, it persists where health systems and social protection fail. For decades, regional situational analyses have documented gaps in coverage, quality, and follow-up within screening programs, especially in countries with territorial inequality and weak health information capacity (12, 15). More recently, PAHO has emphasized regional heterogeneity and the need to strengthen integrated prevention and care pathways that connect screening, diagnosis, treatment, and follow-up with real continuity (13). From the perspective of the cancer continuum, this matters because prevention and early detection are central pillars of control, and because screening only reduces mortality when it is followed by diagnostic confirmation and timely treatment (24).

From this perspective, global targets work only as an integrated package. The 90-70-90 strategy links HPV vaccination, screening, treatment, and follow-up, and it aims to reduce incidence to fewer than 4 per 100,000 by 2030 (16). When one component fails, systems can produce a paradoxical effect: screening detects lesions, but the system does not confirm or treat them, which can drive abandonment, progression, and institutional frustration. Therefore, the chain depends on governance, financing, and legislation. Biomedical evidence alone will not deliver results if the state does not guarantee continuity (16). This is consistent with the broader public health view that vaccination against HPV and hepatitis B, organized screening, and timely treatment are among the most effective and cost-conscious strategies for reducing cancer burden across populations (24).

This framework also supports comparisons across national experiences. Chile stands out for institutionalizing explicit guarantees for cervical cancer through GES/AUGE, with an emphasis on timeliness and financial protection, and for supporting these commitments with standardized clinical guidelines (11, 16). By contrast, countries with weaker fiscal capacity, wider territorial gaps, and limited network governance often struggle to sustain integrated pathways over time (13). In Colombia, epidemiologic and programmatic evidence reinforces the need to analyze differences by territory and by system barriers, both in the natural history of disease and in program performance (8, 33, 34). In El Salvador, studies on screening attendance document social and organizational factors that affect sustained participation even within HPV-based programs, confirming that adopting an evidence-based strategy does not automatically secure consistent use (14).

Across Latin America, clinical time also distributes unequally. Reports describe waits of up to seven months between diagnosis and treatment initiation in contexts such as Mexico and Brazil, with direct effects on timeliness and outcomes (1). These delays interact with economic barriers, including costs for travel, tests, consultations, and treatments, as well as copayments and income loss due to work absences (1). Consequently, cancer costs extend beyond hospitals and can drive impoverishment and household debt through direct spending, indirect income loss, and less visible burdens such as unpaid caregiving time, stress, and domestic reorganization. In centralized systems, these burdens rise because patients must travel and remain away from work and home for longer periods (1, 35).

Public health evidence also shows that risk factors and their costs distribute unequally. In Mexico, the poorest decile reportedly allocates 5.5% of its budget to tobacco consumption, which competes with essential spending on food, health, and education (17). This pattern illustrates a vicious cycle: economic vulnerability increases preventable exposures, and those exposures later manifest as higher disease burden and worse prognosis. Here the prevention lens becomes decisive. Primary prevention reduces exposure before disease appears, and it does so through interventions that are both effective and affordable at population level (24). Comprehensive tobacco control, including taxation, advertising restrictions, and smoking cessation support, lowers tobacco use and reduces the risk of multiple cancers while also generating fiscal benefits for health systems (24). Public policies that promote healthier diets, active transport, environmental protection, and vaccination similarly strengthen cancer prevention by acting on upstream risk conditions rather than waiting for disease to emerge (24). In oral cancer, strongly associated with tobacco and alcohol, researchers have emphasized medical costs and productivity losses, which strengthens the case for regional collaboration in research and control (18).

From an equity standpoint, cancer becomes a health injustice when the probability of living or dying depends on residence, income, employment type, gender, or membership in historically excluded groups. Under these conditions, the problem does not reflect only system inefficiency. It signals a moral and political failure because it shifts risk onto individuals and turns preventable disadvantage into unequal survival (16, 56). In summary, cancer's public health and social relevance in Latin America follows a clear rule: without territorialized and financed policies, regulatory action on commercial and environmental exposures, and networks that ensure real continuity across the cancer continuum, robust information systems, and social protection, scientific evidence will not translate into lives saved, and social inequalities will become survival inequalities.

## 3 Empirical basis and problem diagnosis (evidence, critical bottlenecks, and brief country cases)

Available evidence converges on a central claim: in Latin America and the Caribbean (LAC), health systems often diagnose cancer late and unevenly, not because biomedical knowledge is lacking, but because the real opportunity to prevent, detect, and treat disease depends on how systems function. In this context, failures in financing, governance, network organization, information systems, and workforce capacity turn time into a scarce resource that stratifies access. These failures determine who reaches care in time and who falls behind (1, 16). In practice, cancer progresses while people move through sequential system steps, including appointments, prior authorizations, tests, biopsies, pathology reports, referrals, and treatment initiation. Each step adds delay and cost, and those burdens fall most heavily on people who live far from services, have lower incomes, or rely on segmented systems.

This empirical diagnosis also needs to be read in the post-pandemic context. On May 5, 2023, WHO declared the end of COVID-19 as a Public Health Emergency of International Concern, marking the beginning of a post-pandemic stage in which health systems are expected to assess lessons learned and strengthen resilience (36). Yet the post-COVID transition does not justify narrowing public health to emergency preparedness alone. It requires asking whether promises to “build back better” will actually translate into stronger preventive, promotive, and protective public health capacity capable of sustaining essential services, including cancer control (36). This point matters because the pandemic did not only produce direct mortality from COVID-19; it also generated prolonged disruptions in essential health services, delayed progress in life expectancy, and increased morbidity and mortality from non-COVID causes in many settings (36). For cancer in LAC, this means that current bottlenecks should be understood not only as pre-existing weaknesses but also as part of a broader post-pandemic test of health system resilience.

At the regional level, LAC faces a substantial and heterogeneous cancer burden, with marked cross-country variation in incidence, mortality, and response capacity (37, 38). This heterogeneity reflects more than tumor biology. It also reflects institutional performance, resource availability, continuity of prevention programs, network organization, and the capacity to translate clinical guidelines into accessible services. Cervical cancer illustrates this dynamic particularly well. The literature has long treated cervical cancer as a tracer of inequity because it remains highly preventable and depends on sustained programs to deliver population-level impact (12, 13). When a preventable cancer continues to claim lives, it typically signals system failures, including unstable financing, intermittent screening, loss to follow-up, administrative barriers, and diagnostic bottlenecks.

From a public health perspective, late diagnosis rarely reflects a single event. Instead, it emerges from an accumulated chain of delays. Early detection improves population outcomes only when suspicion leads to confirmation and confirmation leads to treatment within clinically meaningful time. When continuity breaks, detection becomes a promise without impact (39). In LAC, two transitions concentrate the greatest risk: the shift from first contact to diagnostic confirmation and the shift from confirmation to effective treatment initiation. Delays and discontinuities in these transitions often push treatment into later disease stages (1). The post-pandemic period sharpens this concern because resilience depends not only on restoring service volume but also on rebuilding the public health functions that connect surveillance, prevention, care coordination, and equitable access (36).

System fragmentation and bureaucratic gatekeeping function as cross-cutting critical bottlenecks. Fragmentation multiplies administrative checkpoints, prior authorizations, and referrals, and it forces patients and families to manage care pathways on their own. In Colombia, researchers have documented administrative barriers that limit effective access and generate delays even when formal coverage exists (6). Other studies describe access constraints and consequences in Colombian cities (7) and additional barriers in rural areas among subsidized-regime users (10). These patterns intersect with structural obstacles to realizing the right to health, because legal guarantees do not consistently translate into timely care (30).

Diagnostic delays often concentrate in the confirmation phase, particularly in testing, biopsies, and reporting. Pathology sits at the hinge between clinical suspicion and definitive treatment, so pathology capacity and turnaround times shape diagnostic timeliness. When systems fail to secure reasonable timelines, early diagnosis stops operating as a technical standard and becomes an organizational privilege (1). A population-health approach therefore requires health systems to integrate pathology into functional networks rather than restricting it to large hospitals. Centralization concentrates demand in urban centers and reproduces territorial inequities in diagnostic opportunity (29). Global discussions of the pathology workforce also identify distributional deficits and inequities that directly affect diagnostic timelines (53).

Territorial inequality further widens gaps because geography operates as a determinant of access. Distance, travel cost, and travel time condition whether and when people reach care. In Colombia, researchers have described geographic and economic barriers to accessing specialized oncology services in Bogotá, illustrating how concentrated supply can exclude patients through indirect costs (40). At the regional level, research on accessibility and mobility in cancer care, including breast cancer, shows that travel logistics effectively become part of treatment. Patients must travel, secure lodging, miss work, and reschedule visits, and these burdens accumulate, undermining continuity and adherence (35).

In prevention, the core problem often lies less in tool availability than in discontinuous implementation. For cervical cancer, PAHO emphasizes that effective responses require sustained screening, diagnosis, and treatment as an integrated and continuous pathway rather than isolated actions (13). Evidence also highlights the role of information systems in ensuring follow-up, traceability, and monitoring. Without data infrastructure, programs lose women between stages (15). In Colombia, narrative reviews describe barriers to timely detection linked to access, follow-up, and system functioning (9), and analyses based on stakeholder perspectives highlight governance and inter-institutional coordination tensions that disrupt continuity of care pathways (8).

Health system capacity and the effectiveness of rules that govern service delivery complete the set of critical bottlenecks. The availability of imaging, medical oncology, radiotherapy, and essential medicines distributes unevenly across and within countries. As a result, effective pathways often depend on the ability to pay for private tests, travel, or copayments to close system gaps. At the system level, post-pandemic analyses describe health system resilience in LAC as a central challenge that requires stronger stewardship, functional networks, and protected financing to sustain essential services, including oncology care, and to reduce reliance on individual workarounds or payment capacity (19). This argument gains further force from broader post-COVID evidence showing that weak public health capacity was not simply a consequence of the magnitude of the pandemic, but also of chronic underinvestment in public health functions, even in relatively advanced economies (36). In that sense, oncology bottlenecks in LAC should be interpreted within a wider deficit of public health infrastructure, not only within hospital care.

Historically neglected areas also intensify preventable burden, particularly occupational cancer in contexts of high informality. In LAC, labor informality and weak enforcement limit prevention and recognition of work-related cancers, which promotes underdiagnosis and policy inaction (54). For this reason, the region has promoted reference frameworks that treat environment, occupation, and cancer as public health priorities, because exposures do not distribute neutrally and often concentrate among groups with the least protection (25, 28). Estimates of asbestos-related cancer burden in Argentina, Brazil, Colombia, and Mexico reinforce the need for sustained intersectoral policies and effective surveillance (55). More broadly, the post-pandemic agenda underscores that public health institutions must not be reduced to infectious disease control alone. They also have to sustain health promotion, disease prevention, inequality reduction, multisectoral coordination, and research translation, all of which are relevant for cancer control (36).

Brief country cases make these failures tangible. For cervical cancer, PAHO and historical regional analyses document persistent gaps, including unequal screening coverage, program discontinuity, limited follow-up capacity, and unequal access to treatment (12, 13). The critical bottleneck extends beyond implementing screening. Systems must close the full loop by identifying lesions, confirming diagnosis, initiating treatment, and maintaining follow-up, supported by information systems that ensure traceability and prevent losses at each step (13, 15). In Colombia, evidence documents administrative barriers that delay or block effective access (6), constraints in urban settings (7), and additional rural barriers affecting subsidized populations (10). In cervical cancer control, researchers have described obstacles to timely detection and system tensions from stakeholders' perspectives, showing how fragmentation produces discontinuity and clinically inadequate timelines (8, 9). At the same time, Colombia has promoted technical responses through consensus documents and monitoring indicators, including for breast cancer, which signals meaningful policy capacity that still must confront fragmentation and timeliness failures (33, 41).

In Chile, explicit guarantees and standardization through AUGE/GES, supported by clinical guidelines for cervical cancer, can strengthen consistency in care and clarify entitlements (11). However, equity-focused implementation requires the country to secure territorial delivery, timely confirmation, and effective access to treatment nationwide, particularly where resource gaps and capacity concentration persist (13). Comparative analyses of health systems also discuss the role of private-sector involvement and regulatory tensions in Chile and Colombia, which helps explain differences in access and financing arrangements (42). In Nicaragua, PAHO country profiles support context-specific interpretation by linking health system performance to health and socioeconomic indicators, which can clarify why program continuity and installed capacity weaken under constrained conditions (13). In resource-restricted systems, patients often absorb the logistical and financial burden required to sustain care, which intensifies delays and increases the risk of pathway abandonment (1). In El Salvador, a study in the Paracentral Region found that screening attendance depends on organizational factors, access conditions, and prior system experience, confirming that a program's existence does not guarantee its use and that participation declines when systems fail to reduce friction and access costs (14).

The empirical problem in LAC extends beyond cancer as a clinical entity. It includes cancer as shaped by health system performance. Disease progresses while people confront incomplete networks, administrative hurdles, diagnostic delays, centralized capacity, and intermittent prevention. In the post-pandemic stage, this diagnosis also depends on whether countries strengthen the essential public health functions that connect surveillance, governance, financing, workforce development, community participation, equitable service quality, research, and access to technologies (36). These functions have been proposed as minimum requirements for resilient health systems and as a bridge between the health sector and other social sectors, which is especially relevant when cancer outcomes are shaped by administrative, territorial, and socioeconomic conditions as much as by clinical response (36). The public health diagnosis therefore converges: without effective stewardship, adequately financed institutions, information systems that enable follow-up, essential public health functions, and networks that ensure continuity from screening through treatment, preventable disease becomes avoidable mortality.

## 4 Response agenda and debate (proposals and objections)

The response agenda begins with a decisive reframing for Latin America and the Caribbean (LAC): cancer no longer appears only as a clinical event but as a system stress test. When real care trajectories depend on geography, insurance arrangements, or a household's capacity to pay for travel and administrative hurdles, health systems stratify clinical time and distribute it unequally. In that context, organizing the response means intervening where systems fail most consistently: transitions between levels of care, diagnostic bottlenecks, and timely treatment initiation, all of which often dissipate across appointments, prior authorizations, referrals, and waiting lists (1, 16). Public health therefore cannot rely on clinical messaging or isolated campaigns. It requires governance and operational management with measurable targets that track each segment of the patient pathway, from first contact to diagnostic confirmation and from diagnosis to treatment initiation.

A stronger governance lens helps specify how these actions can be articulated and sustained over time. Recent regional analyses emphasize that cancer control in LAC requires organized and continuous efforts rather than isolated measures because structural barriers persist, including health system fragmentation, incomplete universal coverage, inequitable concentration of services, and weak cancer registries (43). In this perspective, governance does not depend on a single institutional model. It depends on each country's ability to organize financing, access, service delivery, and monitoring in a coherent way. This is why universal health coverage appears as a central regional objective, promoted by PAHO, the World Bank, and previous Lancet Oncology commissions, but also why expanded coverage alone cannot guarantee effective access if waiting times, geographic barriers, service availability, and acceptability remain unresolved (43). Governance must therefore move beyond enrollment or financial affiliation and address the concrete bottlenecks that interrupt continuity across the cancer pathway.

At the regional level, this approach translates into fast-track pathways with publicly defined target times that systems monitor and compare across territories. Without operational standards, systems become opaque and normalize delay. This logic aligns with lessons the region already recognizes: when governments fail to secure continuity and coordination, prevention and early diagnosis fragment into episodes that never close the loop. Cervical cancer control in LAC illustrates this pattern through irregular coverage, loss to follow-up, and weak information systems that cannot reliably identify where patients drop out, which helps explain why a highly preventable cancer persists as a marker of inequity (12, 13, 15). In short, guidelines do not define pathways. Systems define pathways when they translate guidelines into accessible, continuous, and auditable services. In this sense, a regional governance framework requires not only technical recommendations but also institutional instruments capable of converting evidence into routinized implementation, protected financing, follow-up, and corrective action (43).

A second pillar of the agenda focuses on what keeps cancer from arriving late to the hospital: sustained prevention with real continuity. For cervical cancer, that means maintaining HPV vaccination and screening with effective follow-up, and ensuring that a suspicious finding moves to confirmation and treatment rather than stalling in backlogs (13, 16). Regional and national evidence suggests that the central challenge rarely lies in technology availability. Instead, systems fail to maintain the full preventive circuit, which requires prevention to function as an integrated pathway rather than as intermittent outreach (12, 13). Implementation research also shows that screening participation responds to concrete points of friction and to users' prior experiences. In El Salvador, for example, attendance remained sensitive to organizational and access factors even within an HPV-based screening program, reinforcing the point that available services do not automatically produce sustained use (14). A governance perspective strengthens this argument by showing that prevention and screening are effective and cost-effective only when policymakers invest in sustainable programs, educational initiatives, and continuity mechanisms that sustain participation and improve population outcomes over time (43).

Within the prevention package, tobacco control cannot remain a peripheral add-on. In a region where systems already operate under heavy demand and fragmentation, reducing avoidable incidence constitutes a structural intervention that protects populations and relieves pressure on care networks. The case becomes more compelling when tobacco spending competes with basic household needs among poorer groups, as documented in Mexico, because preventable exposure can anchor itself in economic vulnerability and then amplify health inequities (17). Sustained prevention therefore saves lives over time and also reshapes system performance in the present by reducing avoidable burden and social costs that hospitals cannot absorb alone. The Latin American and Caribbean Code Against Cancer provides a useful complementary governance framework here. Built by specialists and civil society representatives convened by IARC/WHO and PAHO, it offers 17 evidence-based recommendations adapted to common regional conditions and explicitly distinguishes between recommendations for the general population and policy recommendations for decision makers (44). This distinction matters because it makes clear that preventive behavior cannot depend on individual will alone. Governments must create the structural conditions that allow populations to follow those recommendations through regulation, healthy environments, communication strategies, and intersectoral action (44).

A third pillar involves reorganizing service supply and care networks in ways that reflect a distinctively Latin American constraint: installed capacity tends to concentrate in capitals and referral centers, while peripheral populations face forced mobility to obtain diagnosis and treatment. Networks therefore do not represent an optional enhancement. They provide the minimum condition for real access. In practical terms, this requires regional networks that bring basic diagnostic services closer to patients, including imaging, essential procedures, and pathology, and that ensure effective referral to higher-complexity care through shared standards and inter-institutional coordination (1). It also requires policy and evidence to move together. In oral cancer, for instance, regional collaboration in research and policy offers a mechanism to convert dispersed diagnoses into integrated and sustained responses, especially where risk factors and access barriers overlap (18). Recent regional evidence adds that good practices such as patient navigation and telemedicine can reduce diagnostic and treatment delays and improve continuity of care, although they have not yet been widely incorporated into national cancer plans (43). Effective governance therefore also requires mechanisms for scaling proven innovations from local institutional experiences into national policy.

When this agenda moves to the country level, the logic remains stable, but the leverage points shift with institutional design. Regional experience shows two broad reform paths: some countries, including Peru, Colombia, Chile, Mexico, and Uruguay, have primarily expanded financial coverage through insurance-based arrangements, whereas others such as Guatemala, El Salvador, Honduras, and Paraguay have pursued organizational reforms to strengthen their systems (43). This diversity confirms that governance does not rest on one preferred model, but on whether countries can align financing, organization, and service provision to ensure effective access. Chile provides a salient example of how explicit guarantees and clinical guidelines can organize supply and standardize care, as reflected in the AUGE cervical cancer guideline (11). Even with standards, however, equity depends on territorial implementation, specifically whether systems sustain timely access outside urban nodes and whether regulation prevents public-private tensions from segmenting access or undermining timeliness goals (13, 42). Brazil's 60-day law offers another example of governance through explicit rules, by establishing that the time between diagnosis and treatment should not exceed 60 days; the pilot cited by Barrios et al. showed that median time from diagnosis to treatment decreased from 42 days in 2017 to 37 days in 2018 (43). These experiences suggest that governance becomes tangible when countries define responsibilities, timelines, and monitoring mechanisms that can be enforced and evaluated.

Colombia, by contrast, illustrates the costs of fragmentation and administrative gatekeeping, where administrative barriers delay care even under formal coverage and where these barriers intensify in rural areas and among subsidized populations (6, 7, 10). In cervical cancer, evidence indicates that epidemiologic knowledge alone cannot secure results. Programs require governance and coordination so that they operate as complete pathways rather than disconnected steps (8, 9, 34). Colombia's development of consensus documents and minimum monitoring indicators, including those promoted by Cuenta de Alto Costo, reinforces a central lesson: measurement and risk tracking matter, but they advance equity only when systems connect measurement to real response capacity and continuous care (33, 41). In settings with deeper structural constraints, as suggested by PAHO's national profiles for Nicaragua, the agenda becomes even more foundational: sustain program continuity, strengthen primary care networks, and protect essential diagnostic and treatment capacity so that families do not absorb the logistical and financial burden of care (1, 13). Mexico also illustrates a cautionary lesson: political and institutional restructuring can weaken cancer governance when it disrupts continuity, as seen in the replacement of Seguro Popular by INSABI, a process associated with antineoplastic shortages and continuity problems in care (43). A robust governance framework must therefore protect cancer policy from destabilizing political shifts that interrupt access and quality.

None of these measures will hold if financing remains fragile or discontinuous. In LAC, budget intermittency and weak regulatory implementation often produce supply interruptions, breaks in therapeutic continuity, and deterioration of critical services, failures that carry direct clinical consequences in cancer care (1, 16). The debate therefore must remain explicit: systems need protected financing for timely procurement, maintenance of key services, and uninterrupted treatment, coupled with information systems that track patients, timelines, and outcomes, consistent with earlier recommendations for screening programs in developing contexts (15). Regional governance evidence reinforces that cancer financing must be sufficient, sustained, and distributed across the whole patient journey rather than concentrated only on costly therapeutic innovation. Education, early diagnosis, surgery, radiotherapy, standard chemotherapy, palliative care, and survivorship all require rational and equitable prioritization if systems are to reduce inequalities rather than reproduce them (43). This, in turn, depends on real-time information on procedures, costs, and outcomes, which explains why cancer registries are not peripheral tools but central instruments of governance. High-quality population-based registries, aligned with international standards of comparability, completeness, validity, and timeliness, are indispensable for planning, evaluation, and correction (43). Governance therefore requires not only collecting data but ensuring their quality, integrating them into policy, and sustaining infrastructure, trained personnel, and budget lines that allow evidence to guide decisions (43).

In parallel, having laws does not suffice when enforcement fails. Occupational cancer, historically neglected in the region, demands surveillance, inspection, exposure prevention, and labor protections, because informality and weak oversight foster underdiagnosis and policy inaction, as if exposure did not exist (54). Cancer control thus also unfolds outside the hospital, in workplaces and environments, where state stewardship determines whether societies prevent risk or normalize it. The Code Against Cancer strengthens this point by proposing fiscal, regulatory, educational, and environmental measures that extend governance beyond the health sector, including taxes on tobacco, alcohol, and unhealthy foods and beverages, warning labels, restrictions on advertising and sponsorship, healthy school and community environments, promotion of physical activity and active transport, air quality standards, occupational exposure controls, organized screening and treatment policies, and protection against conflicts of interest (44). This integrated approach makes clear that cancer governance is not limited to clinical care or health financing. It includes regulation of markets, public communication, multisectoral coordination, and fidelity to evidence-based implementation.

Finally, public health reasoning addresses common objections directly. Claims of no resources often translate into no priority, because sustained prevention and early diagnosis avert larger human and financial costs generated by late-stage care (16, 17). Claims that this is clinical overlook the fact that clinical care arrives early or late depending on system performance, which depends on governance, networks, and time management (1). Claims that each country is different remain correct, but they do not remove shared requirements: effective stewardship, clear pathways, financial continuity, functional networks, follow-up data, and implementation capacity. The region already has formal instruments such as national cancer control plans, non-communicable disease plans, and the Latin American and Caribbean Code Against Cancer, but their value depends on whether they are translated into operational capacity, financing, implementation, and sustained monitoring (43, 44). Implementation will vary across territories, capacities, and legal frameworks, but the guiding principle remains equity, because when systems fail, cancer moves beyond disease and becomes avoidable health injustice (1, 54). In that sense, an effective governance agenda for cancer control in LAC rests on a coherent set of pillars: universal coverage with effective access, equitable and protected financing, high-quality data and registries, national plans linked to public policy, and sustained implementation of evidence-based and cost-effective practices that can endure over time (43, 44).

## 5 Closing and public-facing message (call to action plus “one line”)

Cancer in Latin America and the Caribbean cannot remain framed as “bad luck.” When diagnosis arrives late and treatment becomes interrupted or unaffordable, the problem moves beyond the clinic and becomes political and ethical because it exposes structural inequities that distribute timely care as a privilege. The region already has enough evidence to support this claim: the cancer burden is rising, it is unequally distributed, and that inequality translates into avoidable deaths when health systems fail to convert suspicion into confirmation and confirmation into treatment within clinically meaningful windows (1, 37–39, 56).

If bottlenecks emerge along the time chain, policy must treat time as a public good and manage it accordingly. That requires fast-track pathways with verifiable and auditable targets, from first contact to diagnostic confirmation and from diagnosis to treatment initiation, supported by public indicators and continuous monitoring. To sustain this approach, however, governments must do more than improve coordination: they must finance cancer control as a structural public investment rather than as a residual clinical expense. Recent global debates have made this point explicit by reframing cancer financing as infrastructure financing, comparable to energy or transport, and by emphasizing that effective responses require shared governance among governments, industry, philanthropy, and civil society (45). For Latin America and the Caribbean, this means that delivery capacity depends not only on institutional stewardship and operational management, but also on predictable, sustained, and performance-oriented financing capable of supporting referral networks, information systems, and continuity of care across the full pathway (1, 13, 15, 19, 45).

The call to action therefore does not end with demands for “more hospitals.” The region must require governance that enforces timelines, closes territorial gaps, and reduces administrative friction. Ministries of health must translate evidence into delivery capacity through protected budgets, functional referral and counter-referral networks, and performance oversight that rewards continuity and penalizes systematic delays. Legislators must close the gap between law and enforcement by adopting enforceable frameworks and supervision mechanisms because legal text without implementation does not shorten waits or prevent treatment abandonment (1, 56). Yet implementation will remain fragile if financing continues to be volatile, fragmented, or politically subordinated to short-term budget cycles. Current international discussions warn that cancer financing is becoming more difficult precisely when need is increasing, due to declining external support, market volatility, and geopolitical instability, all of which widen the gap between oncologic needs and available resources in low-income and middle-income settings (45). In that context, adopting a clear position on financing is not secondary to cancer control in the region; it is one of its enabling conditions.

Country experiences make this gap tangible. Chile offers a useful example of how explicit guarantees and clinical guidelines for cervical cancer under AUGE/GES can standardize practice and reinforce timely-care rights, provided that the system sustains territorial implementation and maintains capacity beyond major urban centers (11, 42, 46). The point is not to “copy models” but to extract a regional lesson: when a country defines pathways, timelines, and responsibilities and backs those commitments with resources, management, and enforceable financing, it reduces arbitrariness and expands real access. In Colombia, by contrast, evidence has documented the health costs of fragmentation and administrative barriers. Studies of access in cities and rural areas show how paperwork, authorizations, response times, and indirect costs can delay care even under formal coverage, with disproportionate impact on rural and subsidized populations (6, 7, 10, 30). In cervical cancer, reviews and stakeholder-centered studies have identified obstacles to timely detection and coordination failures that break continuity, the basic requirement for prevention to produce population impact (8, 9). Moreover, consensus documents and indicators developed to monitor risk and clinical management in breast cancer show technical capacity, but that capacity requires a system that executes and connects services rather than one that only measures performance (33, 41, 47). Epidemiologically, interpreting cervical cancer burden in Colombia also requires territorially situated analysis that integrates determinants, as classic work has shown (34).

In Central America, inequity also appears when “a program exists” but “a program is used.” In El Salvador, a nested study within an HPV-based screening program documented organizational and experiential factors that reduce sustained attendance, underscoring that systems must reduce frictions to translate nominal coverage into participation (14). In countries facing deeper structural constraints, as suggested by PAHO's national profiles, the agenda must emphasize basic capacity, continuity, and financing even more strongly because patients cannot continue to absorb the logistical burden required to complete care (1, 48, 50). This is also where the financing discussion becomes regionally decisive: Latin America and the Caribbean cannot rely only on traditional funding formulas if they aim to reduce territorial inequalities and sustain cancer control across fragmented systems. Recent proposals point instead to subnational innovative financing, results-based contracting, pooled procurement, and multilateral support as mechanisms that can improve efficiency while aligning cancer priorities with broader health-system reform and universal health coverage goals (45).

For cervical cancer, the regional message must remain explicit: biomedical evidence rarely constitutes the primary constraint; state capacity to sustain the full pathway does. For decades, regional analyses have documented persistent gaps in screening coverage, quality, follow-up, and information systems, weaknesses that help explain why a largely preventable cancer continues to function as a marker of inequity in Latin America (12, 13, 15). The call to action therefore cannot stop at “running campaigns.” It must guarantee HPV vaccination, screening with effective follow-up, and timely treatment as an indivisible package (13, 16). Financing matters here not only in aggregate terms but also in what it chooses to sustain: workforce development across the cancer continuum, cancer registries and electronic health records, medicines and diagnostic equipment, and service-delivery innovations such as task-shifting, teleoncology, and hub-and-spoke models (45). Without financing arrangements that support these functions over time, prevention and early detection remain formally endorsed but operationally weak.

The region must also place regulatory prevention at the core of the agenda. Tobacco control does not function as an optional add-on; it operates as a structural intervention that reduces future incidence and relieves pressure on already fragmented systems. Evidence on tobacco spending among vulnerable groups shows how preventable exposure can compete with basic needs and deepen cycles of disease and impoverishment, reinforcing the political nature of this choice (17). More broadly, financing should not be restricted to high-complexity drugs or technologies alone. Effective cancer control also depends on sustained investment in primary care, community-based services, local implementation research, and the social and regulatory conditions that make prevention and continuity possible (45).

Finally, the closing message should highlight issues the region often postpones: occupational cancer and oral cancer. In economies with high informality and limited enforcement, preventable workplace exposures persist and fuel underdiagnosis and policy inaction. The agenda should therefore elevate prevention, surveillance, and labor protections as core components of cancer control rather than as marginal chapters (28, 54). To sustain real change, Latin America also needs scientific and health collaboration oriented to concrete problems, including applied research networks and policies grounded in local evidence, especially for cancers where prevention, early detection, and coordinated response can reduce burden and mortality, such as oral cancer (1, 18). This also requires confronting the ethical dimension of financing inequity. Recent evidence cited in global discussions shows how little cancer research funding reaches low-income and middle-income countries, underscoring that cancer financing is not only insufficient but also distributively unjust (45, 49, 51). A credible regional agenda must therefore defend equity, transparency, and accountability as governing principles of cancer financing, not as rhetorical add-ons.

Future policy development should also adopt a stronger gender-sensitive and intersectional approach to cancer control across the region. In Latin America, inequities do not affect populations uniformly. They are shaped by the interaction of gender with poverty, rural residence, ethnicity, labor informality, and other forms of structural disadvantage that influence exposure, help-seeking, screening uptake, diagnostic delay, treatment continuity, and survival. A more equitable cancer agenda must therefore move beyond formally universal approaches and incorporate differentiated strategies that identify which groups face the greatest barriers across the cancer continuum and why (52).

This perspective also aligns the regional cancer agenda with the broader commitment to the Sustainable Development Goals, particularly SDG 3 on health and wellbeing. Progress in cancer control in Latin America should be understood not only in terms of treatment expansion, but also in terms of reducing avoidable mortality, strengthening prevention, improving timely diagnosis, and narrowing unjust gaps in access and outcomes across socially and territorially unequal populations.

In Latin America and the Caribbean, cancer rarely kills because of “bad luck”; it kills through avoidable delays, underfinanced pathways, and correctable inequities, and each country can reduce them by turning evidence into enforceable pathways, sustained resources, and public accountability (1, 12, 13, 16, 45).
